# Techno-Economic Analysis of Glycerol Valorization via Catalytic Applications of Sulphonic Acid-Functionalized Copolymer Beads

**DOI:** 10.3389/fchem.2019.00882

**Published:** 2020-01-10

**Authors:** Luma Sh. Al-Saadi, Valentine C. Eze, Adam P. Harvey

**Affiliations:** School of Engineering, Newcastle University, Newcastle upon Tyne, United Kingdom

**Keywords:** glycerol, valorization, sulphonic acid, copolymer beads, solketal

## Abstract

The design of experiments response surface analysis was employed for the first time to study the effect of divinylbenzene (DVB) (20–80 wt. %), diluent (0–100 wt.%), and mixing (200–900 rpm) on the beads' physical properties and on swelling ability. The beads with the highest performances, in terms of mechanical stability, surface area, and swelling ability, were sulphated, and tested in converting glycerol to a valuable product “solketal.” Process options for glycerol valorization to solketal using synthesized sulphonic acid-functionalized styrene-divinylbenzene (ST-DVB-SO_3_H) copolymer beads and techno-economic analysis of the processes have been investigated. Three processes were evaluated: two one-stage processes at 8.5 wt.% catalyst and 50°C, based on either 6:1 acetone to glycerol molar ratio (87% conversion) or 12:1 (98% glycerol to solketal conversion), and a two-stage route (two acetone additions), where ≥98% conversion can be achieved with lower overall acetone use (10:1 acetone to glycerol molar ratio and 50°C). Techno-economic analyses of the three solketal options were performed using Aspen (HYSYS), based on a fixed capacity of 100,000 te/y and 20-years lifetime. The techno-economic analyses showed that the net present values for the solketal process options were $707 M for the two-stage, $384 M for the one-stage at 6:1 acetone to glycerol molar ratio, and $703 M for the one-stage at 12:1 acetone to glycerol molar ratio. The break-even prices for these solketal processes were $2,058/ ton for the one-stage at 12:1 of acetone and two-stage and $2,088/ton for the one-stage at 6:1 of acetone, which is lower than the current price of solketal at $3,000/ton. The two-stage process was found to be the most effective method of glycerol valorization production to solketal.

## Introduction

**Figure 1:**
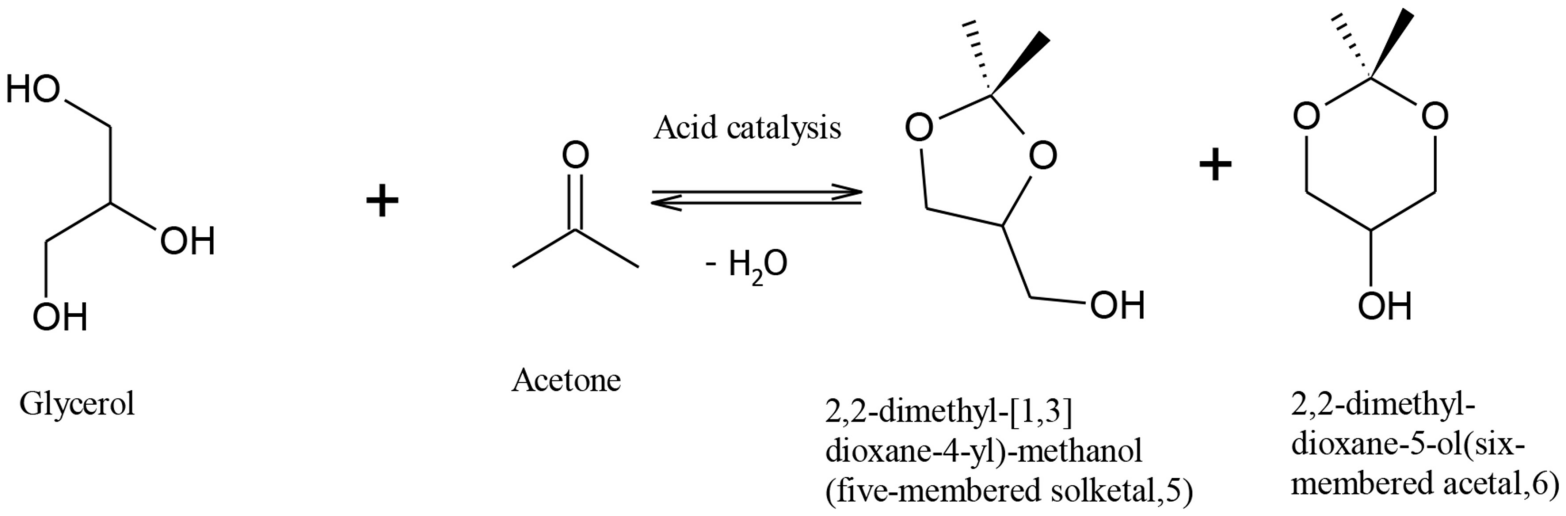
Producing solketal from glycerol (Khayoon and Hameed, 2013).

Rapid depletion of fossil fuel and the harmful effects of its combustion on the environment have motivated the quest to find an economic and effective method to produce renewable fuels with less harmful environmental effects. Bio-fuels, such as biodiesel, have emerged as an environmentally friendly and sustainable substitute to petro-diesel (Demirbas, 2008; Helwani et al., 2009). Biodiesel is most commonly produced via triglyceride transesterification, which produces fatty acid alkyl esters as the main product and crude glycerol as a by-product (Melero et al., 2009; Patil et al., 2009; Kim et al., 2011; Park et al., 2015). It is envisaged that the use of metal oxides, and hydroxide and sulphonic acid-functionalized resin catalysts for biodiesel production, will produce glycerol of high market value, increasing the commercial viability of biodiesel production. The co-production of glycerol in the conventional biodiesel processes has little economic advantage on biodiesel plants as the huge rise in global glycerol production has caused its oversupply, significantly reducing the glycerol price (Rodrigues et al., 2012). Glycerol surplus has increased from 200,000 tons in the year 2003 to over 2 million tons in 2011, and this is predicted to rise to over 6 million tons in 2025 (Ciriminna et al., 2014). Therefore, it is important to find a way to upgrade glycerol into valuable products. Indeed many alternatives have been suggested to utilize glycerol in many fields such as animal food, drugs, cosmetics, tobacco, fuel additives, waste treatment, and production of different chemicals (Knothe et al., 2010; Leoneti et al., 2012; Yang et al., 2012). It also can be used to reduce the free fatty acid content in biodiesel feedstocks, which could help to reduce many of problems related to separation duties and toxicity (Leung et al., 2010). Previous studies have shown that crude glycerol could be converted via a biological process to produce valuable chemicals such as 1,3-propanediol (Mu et al., 2006), citric acid (Papanikolaou et al., 2002), and polyhydroxyalkanoates (Ashby et al., 2004). A promising route for glycerol utilization is reaction with acetone in the presence of an acid catalyst to form 2,2-dimethyl 1,3-dioxalane-4-methanol, also called “solketal” (Mota et al., 2010). The reaction of acetone and glycerol to produce solketal is shown in Figure 1. The reaction is limited by the thermodynamic equilibrium, as complete glycerol conversion cannot be achieved due to water formation (Li et al., 2012; Nandan et al., 2013; Rossa et al., 2017), and long residence time and high amount of acetone are required to overcome this problem (Khayoon and Hameed, 2013). Solketal is a valuable product used as an additive to improve the fuel properties of gasoline and biodiesel, in pharmaceutical industry, and as a plasticizer in the polymer industry (Nanda et al., 2014a).

Conventional processes for solketal production use homogeneous acid catalysts such as p-toluenesulfonic acid (Suriyaprapadilok and Kitiyanan, 2011) and sulfuric acid (Dmitriev et al., 2016), which leads to an increase in the cost of production due to the extra costs of downstream separation of the homogenous catalyst as well as damage to the equipment due to its corrosive ability; thus it cannot be reused. Some homogenous acid catalysts can be easily recovered such as SnF_2_-catalyst (da Silva et al., 2017) and SnCl_2_-catalyst (Menezes et al., 2013), although the presence of chlorides from SnCl_2_ in the reactor can cause corrosion in reactors and pipes. To overcome this problem, heterogeneous catalysts can be used as it is more environmentally friendly than homogenous catalysts; thus, it can be recycled and reused many times, which turn the production process to “green.” Glycerol acetalization was researched using heterogeneous acid catalysis such as Ni–Zr supported on mesoporous activated carbon (Khayoon and Hameed, 2013), zeolite H-BEA (SAR 19) (Rossa et al., 2017), Zr- and Hf-TUD-1 and Sn-MCM-41 (Li et al., 2012), and sulfonic mesostructured silica (Vicente et al., 2010). However, these catalysts have lower rates of reaction than homogeneous catalysts. An alternative is to use an ion-exchange resin supported catalyst, produced from co-polymerization of styrene and divinylbenzene (DVB). Due to the presence of DVB in the structure, the polymeric resin beads swell during the reaction, providing easy access of reactants to the catalytic active sites (Sharma et al., 2011; Boz et al., 2015), such that high reaction rates can be achieved. Ion-exchange resins can be regenerated (Tesser et al., 2010) and reused many times (Huang et al., 2012). Above all, Amberlyst resins, such as Amberlyst 35, exhibit excellent performance in glycerol acetalization (Nanda et al., 2014b). Resin catalysts have been synthesized with variety of physical properties, as the cross linking level has been varied from low for Dowex HCR-W2 and Amberlyst-16 to high for Amberlyst-15 and Amberlyst-35 (Özbay et al., 2008). Different structures (macro-porous and gel-type) were used in the transesterification reaction of biodiesel production; different surface areas, pore diameters, and porosities were produced (Fu and Borges, 2015). The results showed that the physical properties of catalyst such as pores and surface area have more effect on catalytic performance than that of cross-linking level. The catalyst types in order of decreasing activity are A-15 > A-35 >A-16 > Dowex HRC-W2 (Özbay et al., 2008). The authors revealed that the difference between using the abovementioned catalysts as a particle or as a powder is only 10% (Özbay et al., 2008). More study is required to investigate the feasibility of the use of other types of resin structures, such as non-porous and large porous structures.

In the resin synthesis, the organic phase (discrete phase) consists of monomer (i.e., styrene), cross linker (DVB), initiator (benzoyl peroxide), and a porogen, which can be either a solvent, such as toluene, or a non-solvent, such as heptane. The aqueous phase consists of emulsion stabilizer such as hydroxyethyl cellulose, gelatine, and sodium chloride (Gokmen and Du Prez, 2012; Yussof, 2012). The volume of the aqueous to the organic phase is usually fixed above 3:1 (Coutinho et al., 1998).

When toluene was used as a solvent in discrete phase, adding of monomer droplet leads to building a crosslinking continuously. When the crosslinking becomes rigid, it enables to absorb toluene at “a gelation point.” After that the de-swelling (separation) phase occurs. The predominant beads in this case are micro or meso-pores with high surface area and low pore volume (Gokmen and Du Prez, 2012).

**Figure 2:**
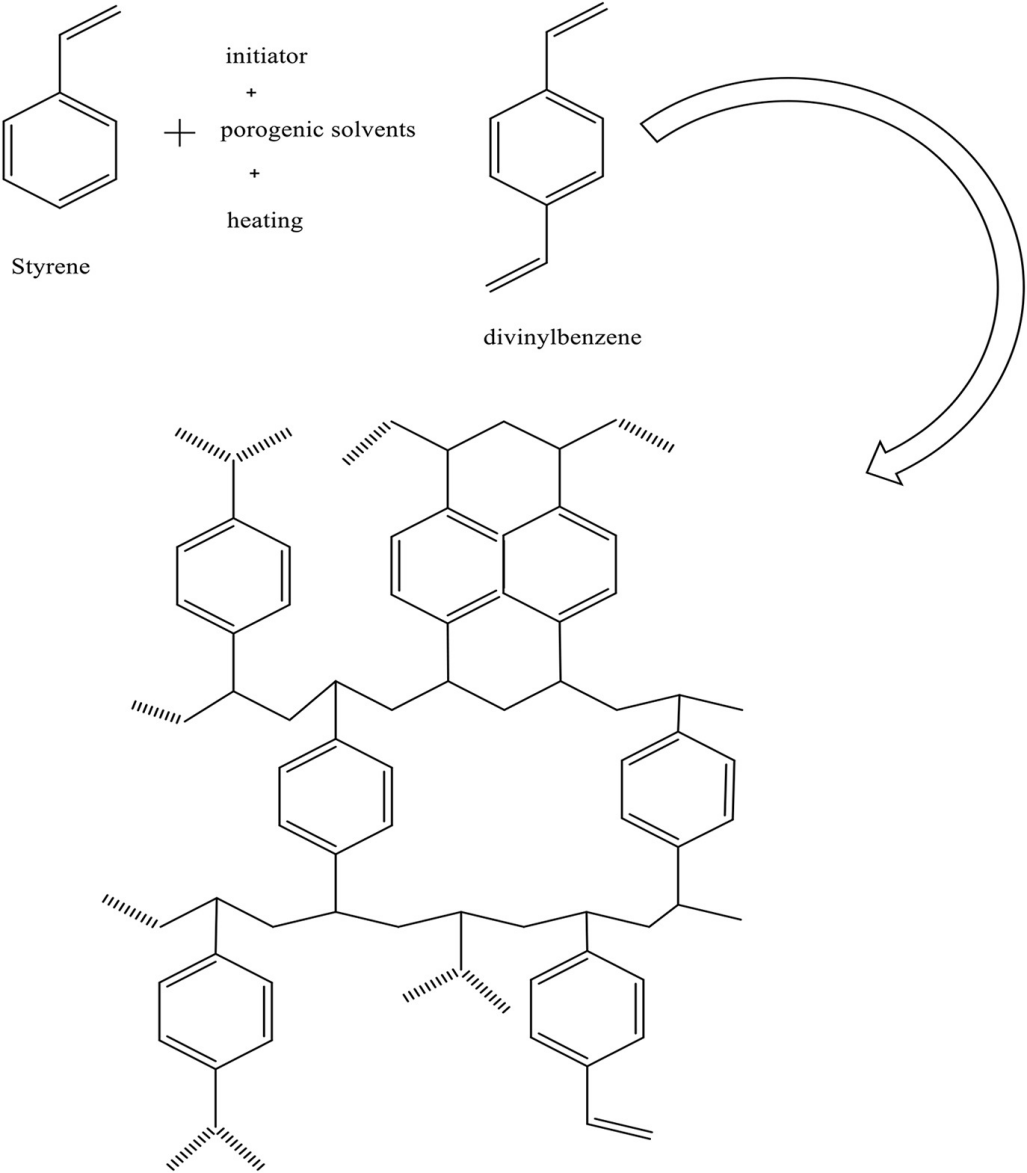
Chemical structure of ST-DVB resin.

Conversely, a non-solvent porogen (such as n-heptane) cannot dissolve or swell the polymer chain, so the separation phase occurs before gelation point. In this case, large pore volumes and very low surface areas of macroporous particles were obtained (Gokmen and Du Prez, 2012) (see the chemical structure of ST-DVB in Figure 2).

However, the styrene-divinylbenzene (ST-DVB) copolymerization must be optimized for the production of copolymers of desirable particle sizes, surface area, and physical properties.

In this study, process options for glycerol valorization to solketal using sulphonic acid-functionalized (ST-DVB-SO_3_H) copolymer beads and techno-economic analysis of the processes were investigated. The solketal processes evaluated were one- and two-stage acetalization processes using synthesized and sulphonic acid-functionalized ST-DVB copolymer beads of desirable properties. The glycerol acetalization process was optimized to overcome the equilibrium limitation and ensure high solketal yields. The ST-DVB copolymer beads were chosen as the sulphonic acid support because the polymerization process could be tailored to achieve copolymer beads of different physical properties. Techno-economic analysis based on Aspen (HYSYS) was applied to evaluate the different process options for glycerol valorization to solketal.

## Materials and Methods

### Materials

The chemicals used in the experiments were styrene (>99%), divinylbenzene (80%), benzoyl peroxide (75%), gelatine from bovine skin (99.5%), heptane (99.9%), toluene (99.9%), 2-hydroxyl ethyl cellulose, vinyl benzene chloride (97%), poly(vinyl alcohol) (>99% hydrolysed), 2,2′-Azobis(2-methylpropionitrile) of 0.2 M in toluene, and sodium chloride (>99%). These chemicals were purchased from Sigma-Aldrich, UK. Amberlyst^TM^ 70, supplied by Dow Chemical Company, Netherlands, was used for catalytic activities comparison, with the synthesized ST-DVB-SO_3_H copolymer beads.

### Synthesis and Characterization of Sulphonic Acid-Functionalized ST-DVB Copolymer Beads

**Figure 3:**
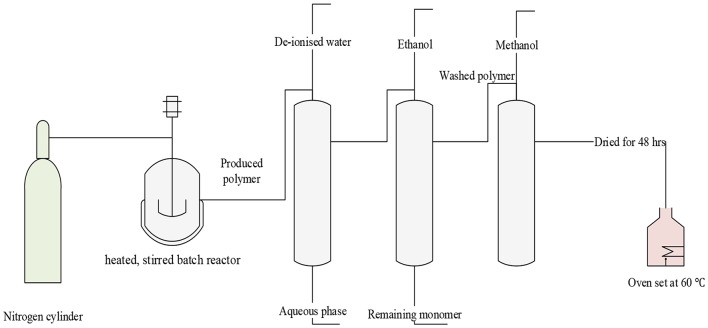
Schematic diagram of copolymerization of styrene and DVB.

The ST-DVB copolymer beads were produced by suspension polymerization of the styrene and divinylbenzene monomers. Copolymerization was carried out in a 500-ml three-necked batch reactor equipped with a heater–stirrer (IKA RCT basic), a reflux condenser, and a nitrogen inlet pipe (Figure 3) was used to carry out the copolymer synthesis. The heater–stirrer was used to control the reaction temperature and the mixing speed.

The copolymerization mixture contained organic phase with monomer compositions of 36 wt.% styrene and 64 wt.% divinylbenzene and the 84 wt.% diluent based on the total monomers solution. The diluent used was a solution of 40:60 toluene-to-heptane volume ratio. The aqueous phase in the suspension polymerization contained de-ionized water with 0.2 wt.% hydroxyl ethyl cellulose, 0.5 wt.% gelatine, and 0.5 wt.% sodium chloride. About 1 wt.% of benzoyl peroxide was added to monomers as an initiator before starting each batch of polymerization.

Copolymerization was conducted using 3:1 aqueous-to-organic phase volume ratio, 90°C temperature, mixing intensity of 900 rpm, and reaction time of 24 h. Preliminary investigations, using design of experiment methodology, showed that these experimental conditions are optimal in achieving the desired ST-DVB copolymer particle sizes and properties. The copolymer beads were washed three times with de-ionized water (until the water became clear) and with ethanol to remove the aqueous phase or any unreacted monomers (Coutinho et al., 1998; Kangwansupamonkon et al., 2002). The ST-DVB beads were also washed with methanol, dried under 60°C for 48 h (Yussof, 2012), and stored for characterization and sulphonic acid functionalization. Design of experiments, using a response surface method, with stepwise analysis was used to investigate the copolymerization conditions.

The styrene-DVB copolymer beads in each polymerization batch were characterized in terms of their morphology, particle size distributions, bead density, surface area, and swelling ratio. Morphology of the resin beads was measured by mounting the samples on aluminum stubs, followed by analysis of their micro-structure in low-vacuum mode at 2 kV, using an environmental scanning electron microscope (Hitachi S2400) equipped with a field emission gun (FEI X30 ESEM-FEG). Sieves with mesh sizes from 2 mm at the upper and 1 mm, 425, 335, 212, and 75 μm at the lower were used. The amount of copolymer beads retained in each sieve and the percentage weights used to calculate the average particle size diameter were obtained. The apparent density of the copolymer beads was determined using Equations 1–3 by gravity method based on the physical properties of the polymers as reported elsewhere (Kangwansupamonkon et al., 2002). The experimental swelling ratio of polymer (*S*) was obtained using Equation 4 by immersing the polymer in excess toluene for 24 h in a tube covered and sealed with aluminum foil (Kangwansupamonkon et al., 2002). The surface area of the beads was calculated based on particle radius and apparent density using Equation 5.

(1) $$\text{Regular particles }=\frac{\text{mass of the polymer}}{\text{volume of occupied container}}$$

(2) $$\text{Small particles or powder }=\frac{\text{mass of the polymer}}{\text{volume of the polymer from mark of cylinder}}$$

(3) $$\text{Irregular particles }=\frac{\text{mass of the polymer}}{\text{volume of displaced water}}$$

(4) $$\text{S}=1+(\frac{{\text{W}}_{\text{s}}}{{\text{W}}_{\text{p}}}-1)\frac{\rho_{\text{p}}}{\rho_{\text{s}}}$$

(5) $$\begin{array}{l} \text{surface area }=\;\frac{\text{area of the particles }(4\pi \;{\text{R}}^{2})}{\text{volume of the particles }(\left(\frac{4}{3}\right)*\pi \;{\text{R}}^{3})}*\left(\frac{1}{\rho_{\text{p}}}\right)\\ \;=\frac{3}{{\text{R}}^{*}\rho_{\text{p}}} \end{array}$$

where *W*_s_ and *W*_p_ are the weights of the fully swollen polymer and the dry polymer, respectively. ρ_*p*_ and ρ_*s*_ are the densities of polymer and solvent, respectively, while *R* represents the average radius of polymer particles.

The dried ST-DVB copolymer beads were functionalized with sulphonic acid sites by treatment with hot, concentrated sulphuric acid. About 5 g of the copolymer beads were transferred into 200 ml concentrated sulphuric acid at 90°C and sulphonated for 160 min. On completion of the sulphonation, the reaction mixture was poured over ice to quench the reaction, and the sulphonated copolymer beads were washed up to four times with de-ionized water, acetone, and methanol and dried at 60°C for 48 h. The ST-DVB-SO_3_H copolymer beads were characterized, and the active sites content was quantified through sulfur content analysis using Elementar Vario Max CNS Analyser. The synthesized ST-DVB-SO_3_H copolymer beads were applied as heterogeneous catalysts for glycerol acetalization to solketal. In the catalytic applications, the spent ST-DVB-SO_3_H copolymer beads were regenerated by treatment with 0.1 M HCl, washing three times with water, and drying at 120°C.

### Glycerol Valorization to Solketal Using the ST-DVB-SO_3_H Catalyst

Heterogeneously catalyzed glycerol acetalization to solketal by catalytic applications of the synthesized ST-DVB-SO_3_H copolymer beads was investigated using a statistical design of experiments, response surface methodology with stepwise analysis. The reaction conditions investigated were acetone to glycerol molar ratios of 2:1–6:1, 1–20 min residence time, reaction temperatures of 30–50°C, fixed catalyst of 8.5 wt.% (based on the glycerol feed), and 800 rpm mixing intensity to ensure that the reactions were kinetically controlled. The glycerol acetalization experiments were performed in a 250-ml batch reactor equipped with a heater–stirrer and reflux condenser. In each experiment, 92.09 g of glycerol and 232.32 g of acetone (for 4:1 acetone to glycerol molar ratio) were charged into the reactor and heated to the reaction temperature. This was followed by adding 7.83 g of the ST-DVB-SO_3_H copolymer catalyst and mixing at 800 rpm.

More experiments were carried out based on the observations from the abovementioned experimental design to investigate other process options for solketal production using one- and two-stage processes at 6:1–12:1 acetone to glycerol molar ratios and 30 min offixed reaction time. Two cases of one-stage glycerol acetalization process were investigated at 6:1 and 12:1 acetone to glycerol molar ratios, using 8.5 wt.% of ST-DVB-SO_3_H copolymer catalyst and at 50°C. The two-stage process was performed using a first-step glycerol acetalization at 10:1 of acetone to glycerol molar ratio, 8.5 wt.% ST-DVB-SO_3_H copolymer catalyst, and 50°C, followed by flash distillation at 10 mbar and 40°C, and a second glycerol acetalization step using fresh acetone. About 1 ml of sample was collected at various time intervals during the reactions using a micropipette, and these were filtered through a 150-μm stainless steel wire mesh in 2-ml vials. All the samples collected were analyzed immediately using gas chromatography.

### Sample Analysis Using Gas Chromatography

The collected samples were analyzed using a 6890 Hewlett Packard gas chromatograph (GC). About 50–80 mg of the homogenized sample was measured into a 2-m GC vial, followed by the addition of 1 ml of 10 mg mL^−1^ of methyl heptadecanoate prepared in 2-propanol. The prepared samples were analyzed using GC by injection of 1 μl of sample with a 5-μl SGE GC syringe. The GC was equipped with a fused silica capillary column of 30 m length, 0.32 mm internal diameter, and film thickness of 0.25 μm. The GC oven temperature program was 120°C for 5 min initially and ramping up from 120 to 260°C at a heating rate of 15°C/min, which was held for another 15 min. The injector and flame ionization detector temperatures were set at 250°C and 260°C, respectively. The glycerol and solketal contents were quantified using a calibration data which were obtained from the response factors of the solutions of glycerol/solketal and methyl heptadecanoate standard prepared in 2-propanol. Glycerol and solketal conversions were calculated using Equations 6, 7, respectively.

(6) $$\text{Glycerol conversion }\left(\%\right)\text{ = }\frac{\text{Glycerol content of the sample }}{\text{Initial glycerol content}}*100$$

(7) $$\begin{array}{r} \text{Solketal yield }\left(\%\right)=\;\frac{\text{Solketal content of the sample }}{\text{Maximum theoretical solketal}}*100 \end{array}$$

### Techno-Economic Analysis of the Glycerol Acetalization Process Options

**Figure 4:**
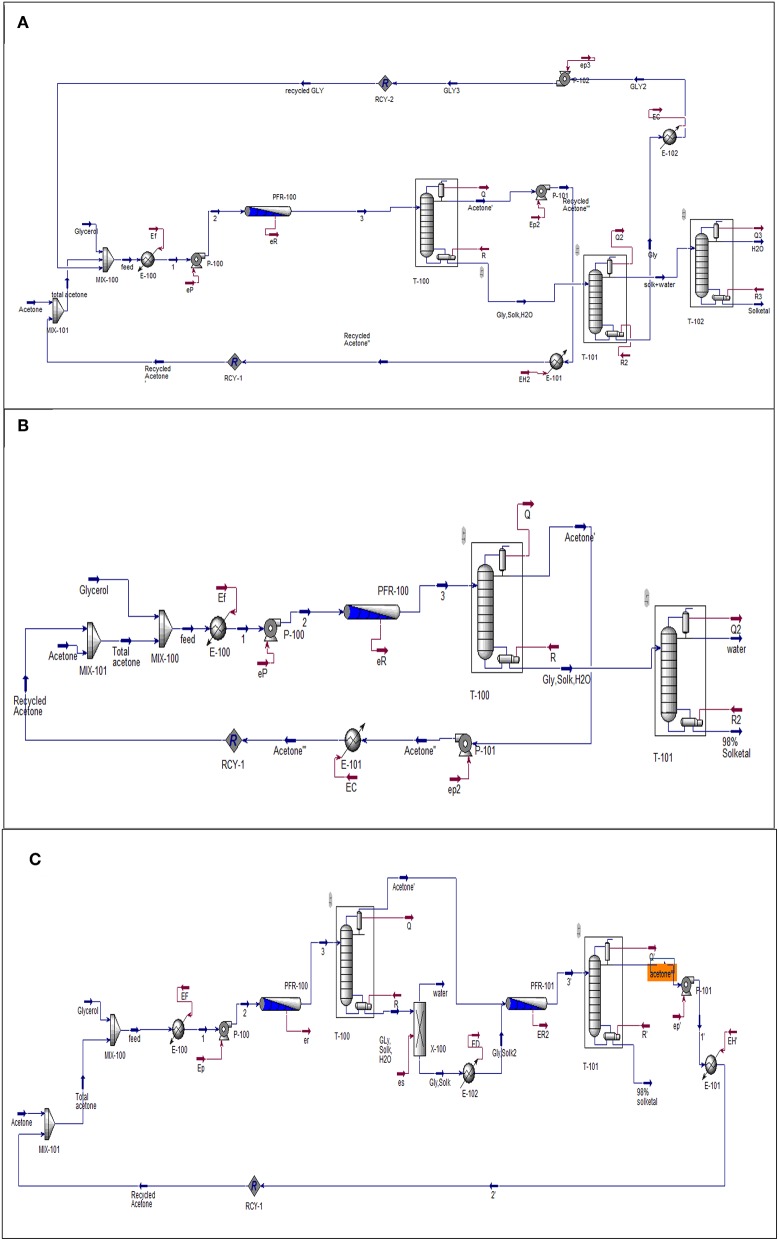
Flowsheet diagram of glycerol acetalization *reaction*. **(A)** One-stage process (low acetone), **(B)** one-stage process (high acetone), and **(C)** two-stage process. Gly is glycerol, Solk is solketal, Mix is mixture, E is heat exchanger; T-100, T-101, and T-102 are distillation columns, PFR is a plug flow reactor. P-100, P-101, and P-102 and pumps and all the streams in red lines represent the energy required by the equipment.

Aspen (HYSYS) was used to simulate the three process options that have been investigated to produce solketal from the heterogeneously catalyzed acetalization of glycerol in the presence of the synthesized ST-DVB-SO_3_H copolymer catalyst. In this study, the process plants were simulated at fixed capacity of 100,000 *tons/year*, 20 years lifetime, and fixed residence time of 30 min. The flowsheet in Figure 4 was proposed for all three solketal process plants. The one-stage solketal process plants operated at 8.5 wt.% ST-DVB-SO_3_H copolymer catalyst and 50°C, based on either 6:1 with 87% glycerol to solketal yield (based on the experimental data; Figure 4A) or 12:1 of acetone to glycerol molar ratio with 98% glycerol to solketal yield (based on the experimental data; shown in Figure 4B). Figure 4C shows the third solketal process, which was based on glycerol acetalization at 10:1 of acetone to glycerol molar ratio, 8.5 wt.% ST-DVB-SO3H copolymer catalyst, and 50°C with 84% glycerol to solketal yield in the first step (based on the experimental data), followed by removal of reactively formed water, and another glycerol acetalization step at the same reaction conditions to achieve a total of 98% glycerol to solketal yield (based on the experimental data). The NRTL model was chosen to model this process (Sakdasri et al., 2018). The economic viability of these glycerol acetalization processes were evaluated based on the 20-years net present values (NPV) and the sensitivities of the plants' profit margins to the fluctuations in the reactant and product market prices.

The sensitivity analyses were determined by varying the price of the reactants and the products from −50 to +50% and calculating the effect of price changing on the net present value. The NPV is the cumulative discounted free cash at the end of the project (in this study, it was based on 20 years of operation and 1 year of construction); it was calculated using Equation 8. The trends in reactant and product prices lead to changes in the break-even price of the produced biodiesel, which is measured as the minimum selling price of biodiesel to achieve positive NPV.

(8) $$\begin{array}{r} NPV=\overset{T}{\underset{t=1}{\sum }}\frac{C_{in,t}}{{\left(1+r\right)}^{t}}-C_{out} \end{array}$$

where *C*_in_, _*t*_ represents net cash inflow in time *t, C*_out_ represents initial capital expenditure, and *r* is the discount rate.

The economic analysis of the various biodiesel processes in this study was based on the following assumptions:

- RSO feed of 100,000 tons/years was chosen as a case study
- Plant lifetime of 20 years
- Pump efficiency of 75%
- Equipment purchase costs from the HYSYS database
- The total investment cost was calculated based on the investment cost required to build the plant in addition to operating cost.

## Results and Discussion

### Synthesis, Functionalization, and Characterization of the Copolymer Beads

Table 1 shows the properties of the copolymer beads produced at various process conditions. The data were analyzed using the stepwise response surface method, implemented in Minitab 17. This analysis was applied to evaluate the effects of the operating parameters and generate empirical models for the average particle size, surface area, and swelling ratio. The experiments carried out at 200 rpm were ignored as no particles were formed at that mixing condition. All of the experiments were repeated twice.

**Table 1:** Characteristics of ST-DVB copolymer beads produced at different reaction conditions.

**Run number**	**DVB** **wt.%**	**Diluent** **(wt.%)**	**Mixing (rpm)**	**Average particle diameter (mm)**	**Swelling ratio**	**Surface area** **(m^2^/gm)**	**Particle shape**
1	20	50	900	0.753	491.0	132	Spherical
2	20	50	200	−	133.0	−	No particles
3	20	0	550	0.856	337.7	76	Spherical
4	20	100	550	0.69	264.5	103	Spherical
5	50	50	550	0.416	174.0	240	Spherical
6	50	0	900	0.493	288.0	233	Spherical
7	50	100	900	0.393	455.0	237	Spherical
8	80	50	200	−	391.0	−	No particles
9	80	100	550	1	593.0	22	Spherical
10	80	0	550	1.78	1082	42	Spherical

The average particle size, the swelling ratio, and the surface area for the styrene-DVB copolymer beads are predicted by the empirical models in Equations 9–11, respectively.

Particle size (mm) = 1.9734–0.05624 X−0.00547 Y−0.000523 Z + 0.000718 X^2^-0.000102 X^*^Y+ 0.000011 Y^*^Z.

(9) $$\begin{array}{r} R^{2}=96.3\% \end{array}$$

Swelling ratio = 3,150–18.89 X−56.71 Y−6.221 Z−0.1711 X^2^ + 0.05423 Y^2^ + 0.000576 Z^2^-1.456 X^*^Y + 0.08621 X^*^Z + 0.1396 Y^*^Z.

(10) $$\begin{array}{r} R^{2}=95.3\% \end{array}$$

Surface area^*^10^−5^(m^2^/gm) = −1.961 + 0.1625 X + 0.01182 Y + 0.000213^*^ Z−0.001611 X^2^-0.000096 Y^2^-0.000149 X^*^Y+ 0.000006 Y^*^Z

(11) $$\begin{array}{r} R^{2}=99\% \end{array}$$

where *X* is the weight percent of DVB, *Y* is the weight percent of the diluent, and *Z* is the mixing speed in revolution per minute.

**Figure 5:**
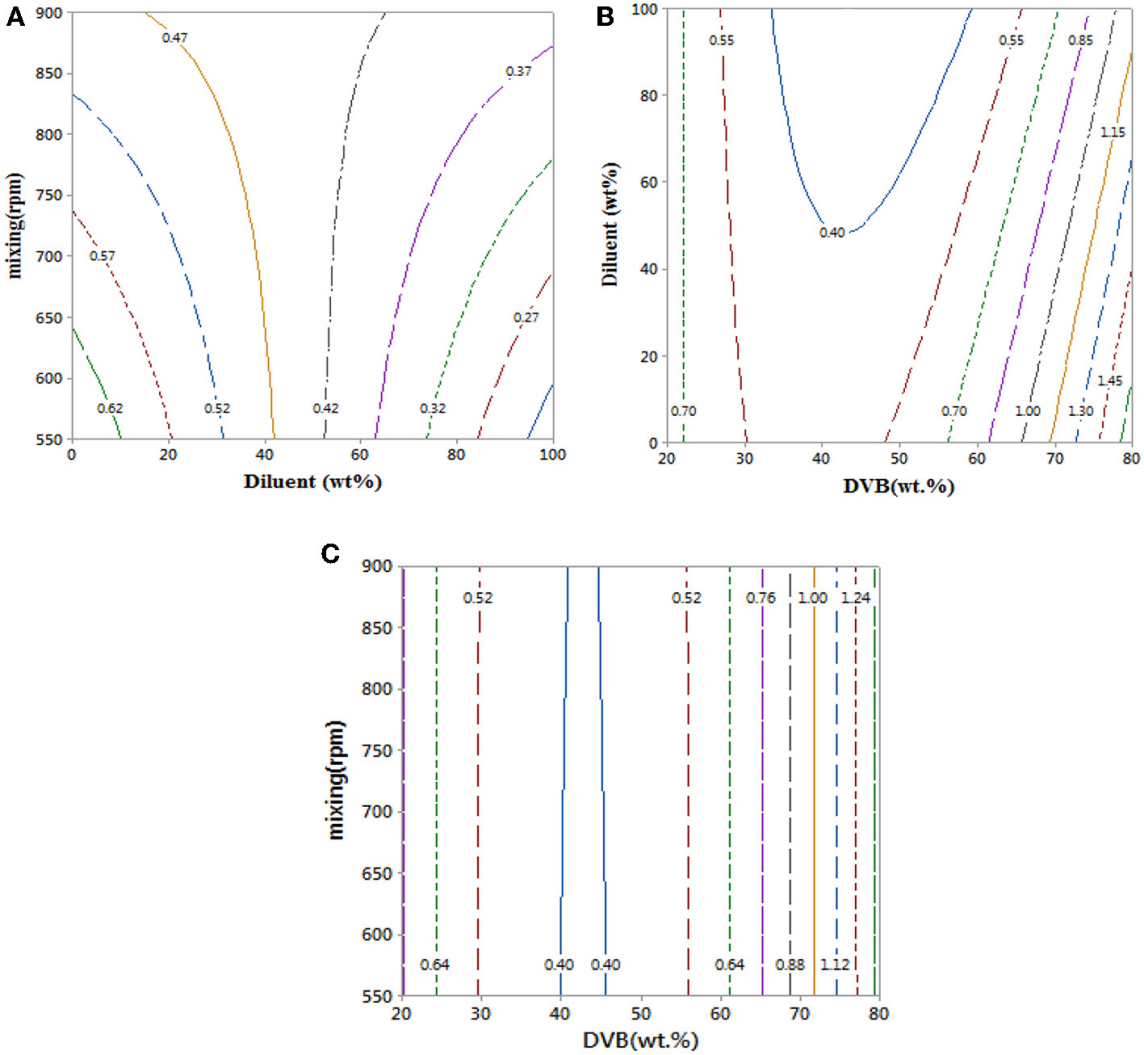
**(A–C)** Contour plots showing the effect of reaction parameters on bead particle sizes. At hold values of DVB 50 wt.%, 50 wt.% of diluent: monomer (wt.%), and 725 rpm.

Contour plots of the experimental data in Figure 5 and data in Table 1 show that mixing has a clear effect on particle size. This is because mixing is required to overcome the reactants' viscosities to generate small monomer beads. There was no substantial change in average particle size when increasing the value of diluent from 0 to 50% as the average particle sizes that obtained from empirical equation were ranged (0.42–0.62 mm) (see Figure 5A), which is consistent with what has been reported elsewhere (Kangwansupamonkon et al., 2002).

**Figure 6:**
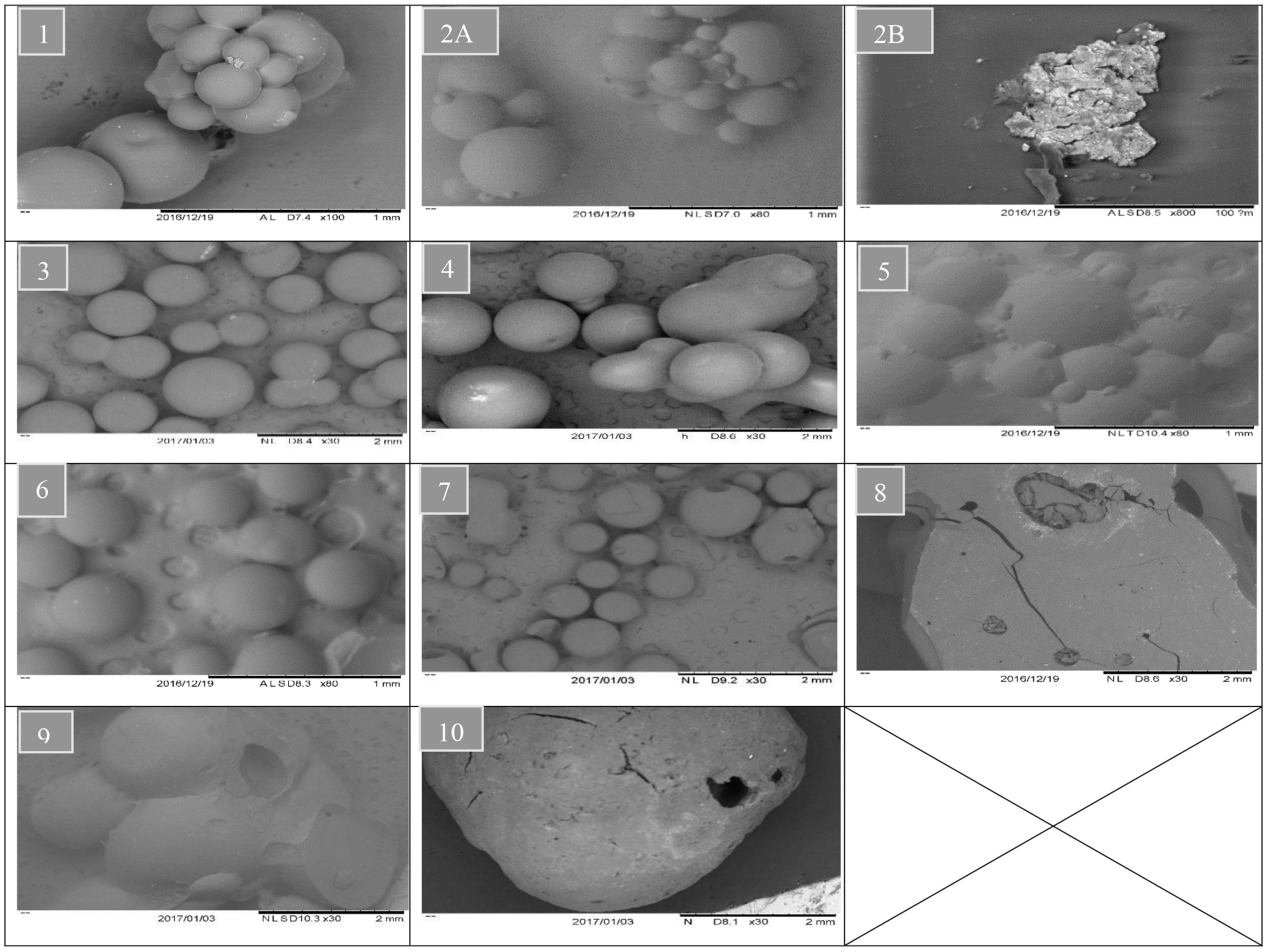
SEM analysis of different samples. The sample number indicating runs number in Table 1.

Whereas increasing the diluent above 50 wt.% can lead to synthesis of styrene-DVB particles of smaller sizes due to substantial reduction in solution viscosity [see entries (3, 4) and (6, 7) in Table 1 and Figure 6]. To obtain small particle size (≤0.3 mm) at high diluent (80–100%), little mixing is required as shown in Figure 5A as increasing the diluent leads to reduced concentrations. For example, at diluent (80–100%), a particle size of ≤0.27 mm can be obtained at only 550–650 rpm of mixing intensity.

Increasing the DVB contents led to increased solution viscosity and consequent formation of beads of large average size [as can be observed in entries (4, 9) in Table 1], such that high diluent amount and high mixing intensity are required to overcome solution density to get small particle size (high surface area). However, because the effect of DVB on the particle size was more than that of mixing and diluent on the bead size, the interaction between the mixing, diluent, and DVB is not significant as shown in Figures 5B,C.

It was observed that at DVB concentrations of 20–30%, the particle size ranged from 0.52 to 0.7 mm at 0–100 wt.% of diluent and 550–900 rpm of mixing intensity as shown in Figures 5B,C. However, under the same operating conditions at 40–50% of DVB, the particle sizes reduced to 0.4 mm; this is because at very small amounts of DVB (20–30 wt.%), the crosslinking between the particles is weak; therefore, the particles agglomerated to form larger particles than at higher DVBs (40–50 wt.%). They increased again to ≥0.85 mm at high crosslinking beads of DVB (60–80 wt.%) due to the increasing of solution viscosity.

It has been reported that high DVB and diluent contents led to the formation of highly cross-linked styrene-DVB particles, whereas the level of crosslinking was lower in the copolymer particles produced at lower DVB contents (Kangwansupamonkon et al., 2002).

In summary, increasing the DVB content leads to higher degrees of crosslinking in the styrene-DVB particles and higher viscosity of the polymerization solutions. Therefore, to control the particle sizes and to synthesis styrene-DVB copolymer beads with substantial degrees of crosslinking, the mixing speed, DVB, and diluent contents must be controlled. For instance, high mixing and diluent content were required to produce small particle sizes in the range of 0.3–0.6 mm, while low diluent contents and mild mixing produced large particle sizes >1 mm.

Depending on the particle size, the surface area of the bead varied. As can be seen in Table 1, the surface area of the beads is inversely proportional to the particle diameters, as when the particle diameter decreased from 0.75 mm (run 1) to 0.39 mm (run 7) for example, the surface area increased from 132 to 237 m^2^/gm, respectively; so to obtain high surface area particles, small bead diameters are required.

**Figure 7:**
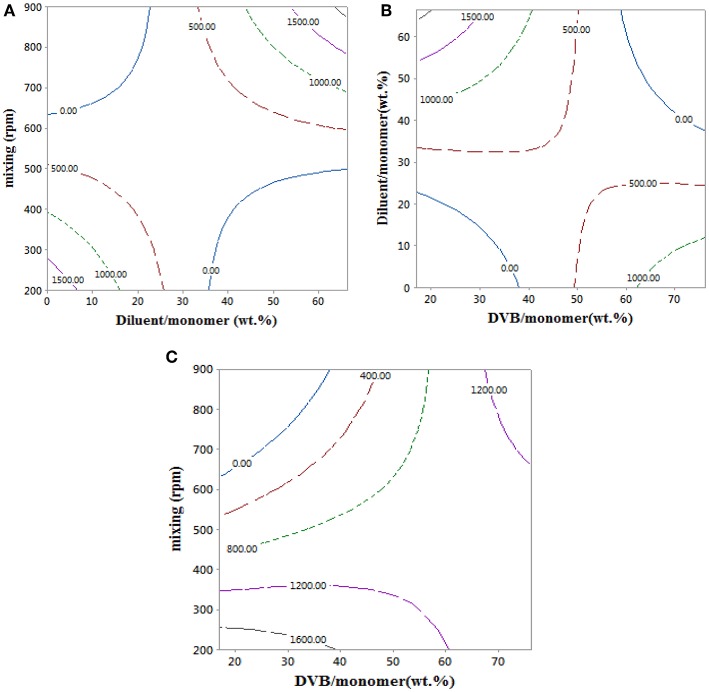
Contour plots showing the effect of reaction parameters on swelling ratio at 0 wt.% of diluent/monomer, 17 wt.% of DVB/monomer, and 900 rpm.

An empirical model for the styrene-DVB beads swelling ratio in Equation 10 was used to obtain the contour plots in Figure 7, which shows the effects of the copolymer bead synthesis conditions on their swelling ratio. Swelling ratio in the copolymer beads take place via solvation of the network chain and filling of pores (Kangwansupamonkon et al., 2002).

The swelling ratio of the copolymer particles increased with mixing intensity. This is attributed to the formation of styrene-DVB beads of small particle sizes at high mixing intensity, which translates to higher particle surface area exposed to toluene and consequently higher swelling ratio as shown in Figures 7A,C. At low DVB content (20 wt.%), increasing the amount of diluent reduces the solution viscosity, such that porous copolymer beads of small size could be achieved, even at mild mixing conditions, and consequently higher values of swelling ratio were achieved as shown in Figure 7B. When DVB contents of more than 50% were used, the copolymer beads produced were highly cross-linked, leading to low porosity (Kangwansupamonkon et al., 2002), which explains the lower swelling ratio of the styrene-DVB copolymers at >50% content of DVB in Figures 7B,C.

At high DVB content (80 wt.%), the particles are non-porous (Kangwansupamonkon et al., 2002), so the swelling ratio is small, but when no diluent was used the particles tended to be fragile due to their high porosity as shown in Figure 6 (3, 6, 10), which increased the swelling ratio (Figure 7B). At zero diluent, the polymer had a high porosity (Durie et al., 2002), so the swelling ratio was high even at high crosslinking (Figure 7C). This decreased with increasing diluent ratio >30 wt.% and increased again at high mixing speed due to the formation of copolymer beads of small particle sizes as shown in Figure 7A. At high mixing agitation, small particle sizes are formed, such that small swelling ratio can be achieved and increased again at diluent (>30 wt.%) and low cross-linking particle (DVB < 60 wt.%) as shown in Figures 7A,C.

The properties of the copolymer beads produced for the copolymerization using monomer compositions of 36 wt.% styrene and 64 wt.% divinylbenzene, 84 wt.% diluent based on the total monomers' solution, 90°C temperature, 900 rpm mixing intensity, and 24 h reaction time. The mean particle diameter for the ST-DVB-SO_3_H beads using sieves was 340 μm. Elemental analysis of the ST-DVB-SO_3_H copolymer beads showed that it contained 8.63 wt.% of sulfur, corresponding to 2.64 mmol -SO_3_H per gram.

### Catalytic Activity and Solketal Productions Using the ST-DVB-SO_3_H Catalyst

**Figure 8:**
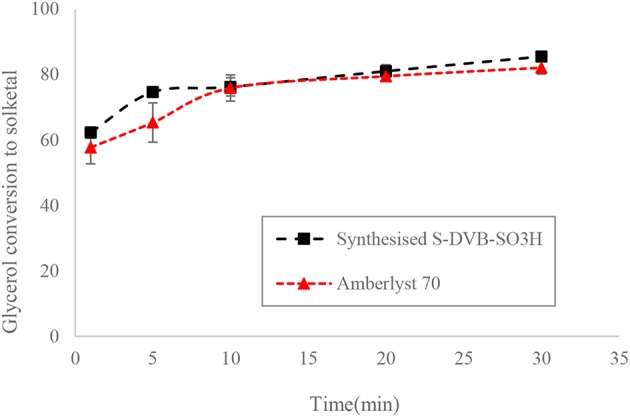
Glycerol conversion to solketal at 6:1 of acetone to glycerol molar ratio, 8 wt.% of catalyst: glycerol and 50°C.

The ST-DVB-SO_3_H copolymer beads were characterized for their physical and chemical properties. The ST-DVB-SO_3_H beads were spherical with 340 μm mean particle diameter. Elemental analysis of the ST-DVB-SO_3_H copolymer beads showed that it contained 8.63 wt.% of sulfur, corresponding to 2.64 mmol -SO_3_H per gram of copolymer beads. The active site density of the synthesized ST-DVB-SO_3_H copolymer catalyst beads was similar to that measured for the Amberlyst^TM^ 70, which has an acidic capacity of 2.59 mmol H^+^/g as measured by sulfur content elemental analysis (Eze and Harvey, 2018). It was also observed that the ST-DVB-SO_3_H had similar catalytic activity as the Amberlyst^TM^ 70 (as shown in Figure 8).

Results for the design of experiment investigations of solketal production using the ST-DVB-SO_3_H copolymer beads for glycerol acetalization at 2:1–6:1 acetone to glycerol molar ratio, residence times of 1–20 min, 8.5 wt.% of catalyst, and 30–50°C reaction temperatures, are shown in Table 2. The experimental data in Table 2 were analyzed by stepwise response surface methods using a Minitab 17 statistical software to obtain an empirical model for glycerol conversion to solketal (*X*; shown in Equation 12), where MR is the acetone-to-glycerol molar ratio, *t* is reaction time (min), and *T* is reaction temperature in °C. Experimental error for the data in Table 1 was ±2%, and this was obtained from the three repeated experiments at run numbers 10, 11, and 15.

(12) $$\text{X}(\%)=25.43+1.325\text{ MR}+0.391\text{ t}+0.637\text{ T}+0.1615\;{\text{MR}}^{*}\text{t}$$

**Table 2:** Experimental data for glycerol conversions to solketal.

**Run** **number**	**Acetone/** **glycerol molar ratio**	**Residence time (min)**	**Temperature (°C)**	**Glycerol conversion to solketal (%)**	
				**Experimental values**	**Predicted** **values**
1	4	20	50	81	83.3
2	6	20	40	87	86
3	4	1	50	62	63.6
4	6	10.5	50	83	79.5
5	2	20	40	70.9	67.8
6	2	10.5	30	54.4	54.7
7	6	10.5	30	66.7	66.7
8	4	1	30	55	50.9
9	6	1	40	56.4	60
10	4	10.5	40	71.65	67
11	4	10.5	40	69.96	67
12	4	20	30	66.3	70.6
13	2	1	40	52.5	54.3
14	2	10.5	50	67.2	67.4
15	4	10.5	40	67.8	67

**Figure 9:**
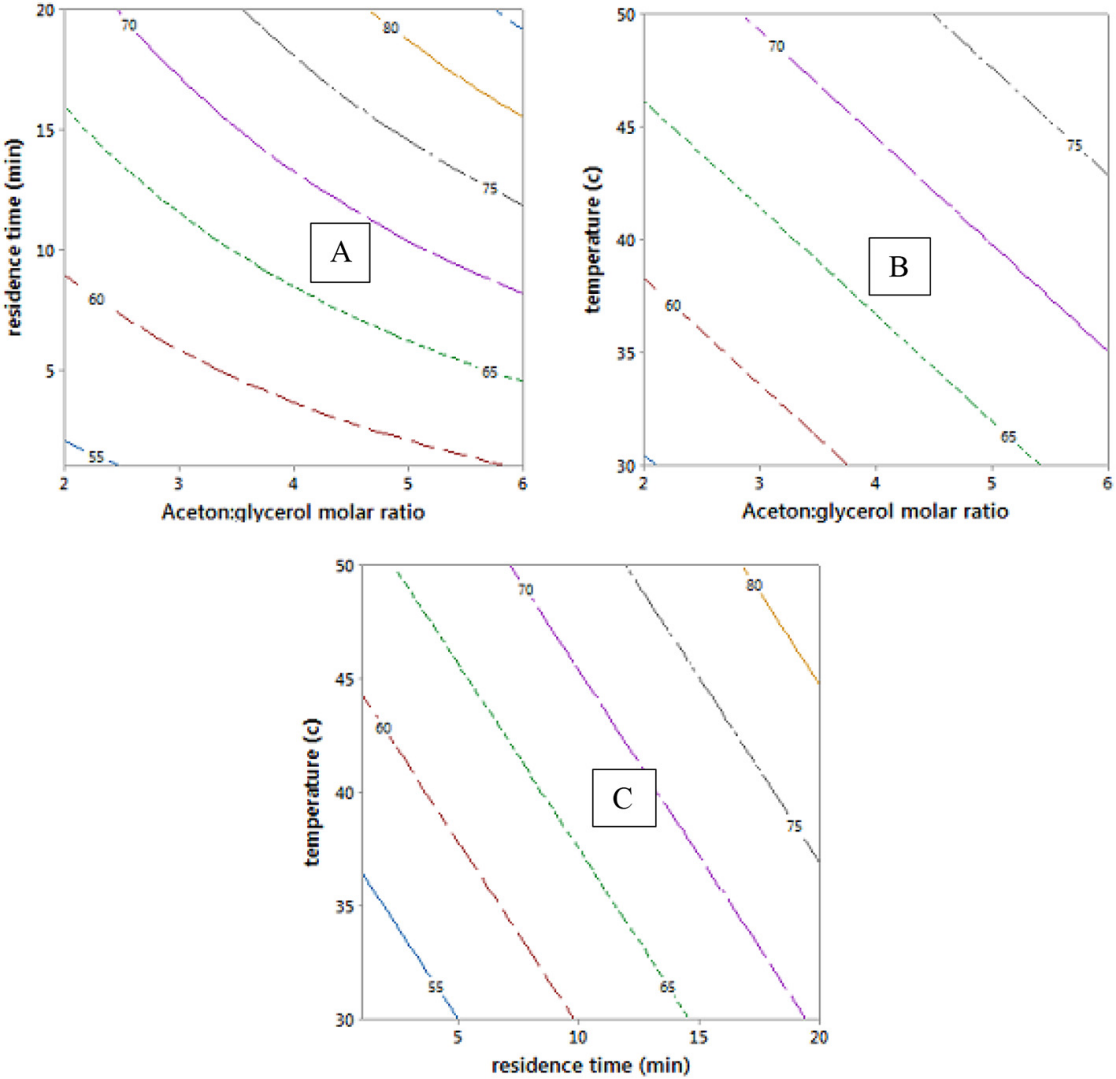
Contour plots of glycerol conversion to solketal at hold values of 4:1 of acetone to oil molar ratio, 10.5 min of residence time, and 40°C.

The empirical model in Equation 12 was used to generate the contour plots in Figure 9, showing the effects of the process variables. It is clear from Equation 12 that acetone to glycerol molar ratio has the strongest positive effect on the conversion of glycerol to solketal as the acetone to glycerol ratio can enhance the forward reaction (Nanda et al., 2014b) (see Figures 9A,B). A steady increase in the conversion of solketal can be observed from Figures 9B,C, when the reaction temperature increased. This is attributed to both the increasing inherent rate of reaction (Khayoon and Hameed, 2013) and decreased glycerol viscosity at high temperature. Similarly, high residence time was required to obtaine high glycerol to solketal conversion as shown in Figure 9C. The empirical equation for glycerol conversions to solketal was experimentally validated at the process conditions, and the results showed that the experimental conversions agreed well with predicted values.

Glycerol conversion to solketal is limited by thermodynamic equilibrium. This, for example, limits conversion to 81% at 4:1 of acetone to glycerol molar ratio and 50°C and to 87% at 6:1 and 40°C in the presence of 8.5 wt.% of ST-DVB SO_3_H (as shown in Table 1). Other studies showed that solketal yield was 63.21% at 4:1 and 60°C with 1 wt.% of zeolite H-BEA (SAR 19) catalyst (Rossa et al., 2017) and 81% at 6:1, 70°C, and 5 wt.% of sulfonated carbon–silica catalyst after 30 min (Nandan et al., 2013). Clearly, the process conditions must be adjusted to overcome the equilibrium limitations. There is a need to increase solketal production from glycerol above the equilibrium conversions in the range of 75–80%, commonly obtained in a single-stage process in the industry (Dmitriev et al., 2016).

**Figure 10:**
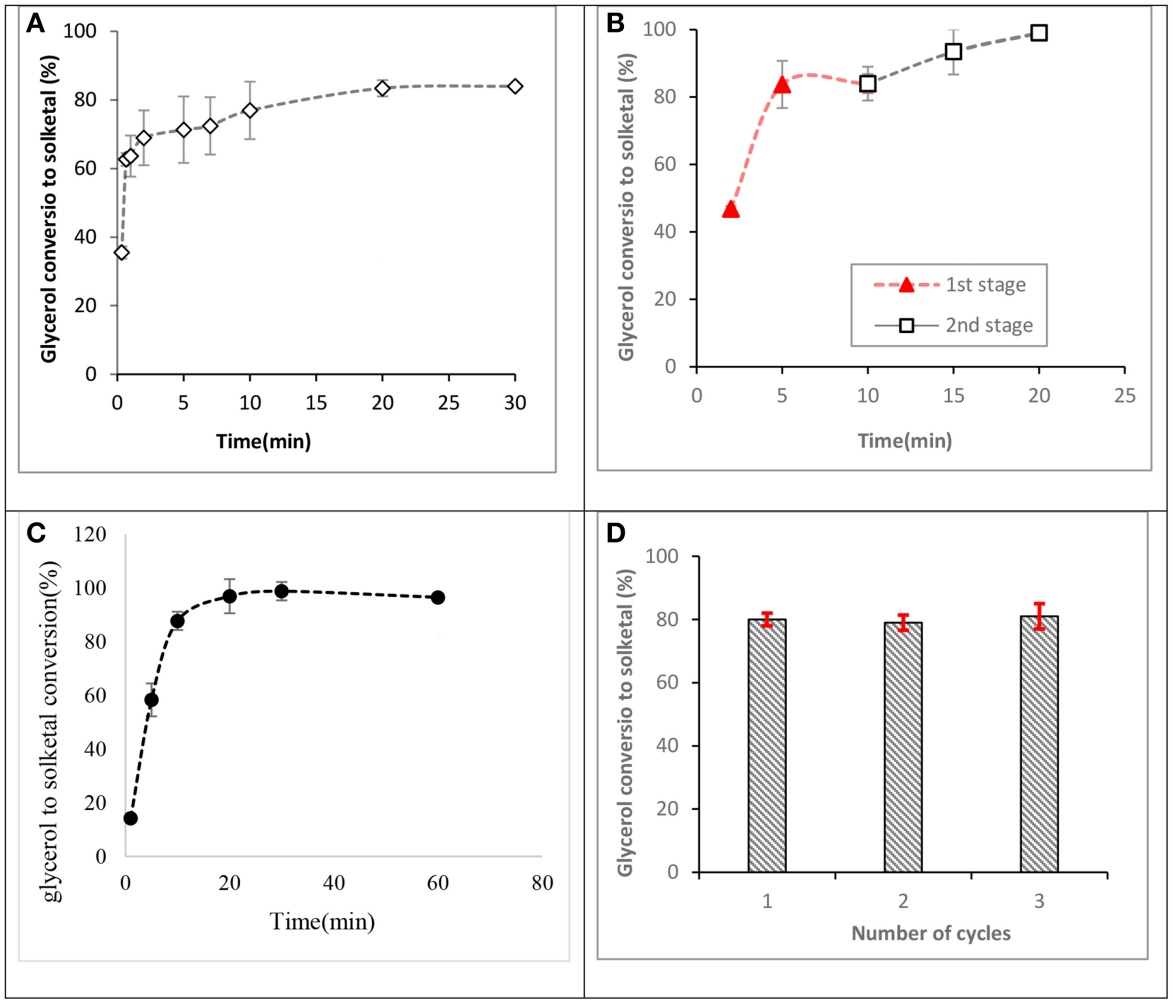
Glycerol conversions to solketal at 50°C. **(A)** Reaction profile for a fresh catalyst at 7:1 of acetone to glycerol molar ratio and 8.5 wt.% of catalyst to glycerol, **(B)** a two-stage process at 10:1 of acetone to glycerol molar ratio and 8.5 wt.% of ST-DVB-SO3H catalyst to glycerol, **(C)** 12:1 of acetone to glycerol molar ratio and 8.5 wt.% of ST-DVB-SO3H catalyst, and **(D)** reusability of the styrene-DVB copolymer catalyst at 12:1 of acetone to glycerol molar ratio, and 5 wt.% of ST-DVB-SO_3_H catalyst to glycerol at 2 h.

Investigations of glycerol acetalization using the synthesized ST-DVB-SO3H catalyst indicate that high glycerol conversion to solketal could be obtained in a few minutes, with maximum conversion occurring in the range 2–20 min at 7:1 of acetone to glycerol molar ratio and 50°C as shown in Figure 10A, contrary to existing reports that equilibrium glycerol conversion to solketal requires residence times over 6 h at 1:2 of acetone to glycerol molar ratio and 80°C when mesoporous silicate Hf-TUD-1 catalyst was used (Li et al., 2012) and 30 min at 6:1 of acetone to glycerol molar ratio and 70°C when sulfonic acid-modified mesostructured silica (Ar-SBA-15) catalyst was used (Vicente et al., 2010). This indicated the catalytic activity of St-DVB catalyst for this reaction.

Previous studies have reported that 4:1 of acetone to glycerol molar ratio was an optimum condition to achieve ≈ 75% of glycerol conversion to solketal after 2 h using SnCl_2_ catalyst (Menezes et al., 2013) and 80% conversion after 1 h with amphiphilic catalysts (Souza et al., 2015). In this work, it was found that by increasing the acetone to glycerol molar ratio to 12:1, complete conversion (up to 98% of glycerol conversion) was obtained in only 30 min (as shown in Figure 10C). This is because, in addition to the high catalysis rate by the ST-DVB-SO_3_H catalyst, the use of acetone in excess shifts the thermodynamic equilibrium toward greater solketal formation, in accordance with Le Chatelier's principle.

Another effective route for achieving higher glycerol to solketal conversions was found to be through a two-stage acetalization process, which requires shorter reaction times under an economic reaction condition. The two-stage route applied a reaction at 10:1 acetone to glycerol molar ratio, with 8.5 wt.% ST-DVB-SO_3_H copolymer catalyst, and 50°C for 10 min in the first step, followed by catalyst separation and drying in vacuum distillation at 40°C and 10 mbar for 1 h, and a second step reaction with the same amount of acetone of 10:1 molar ratio was added, 8.5 wt.% catalyst, and 50°C for another 10 min. The two-stage process achieved about 99% glycerol to solketal conversion after a total reaction time of 20 min (as shown in Figure 10B). A multi-stage batch process has been used to slightly drive forward glycerol conversion to solketal at reaction temperature of 70°C and 5 wt.% of propyl sulphonic acid-functionalized mesostructured silica catalyst, where 89.5% of glycerol conversion was obtained after the third step (Vicente et al., 2010). The lower solketal conversion in that study could be attributed to the higher temperature of 70°C, which is well above the acetone boiling point, such that excessive loss of acetone during the reaction resulted in lower equilibrium solketal yield.

Figure 10D shows the results for the regeneration and reusability study for the ST-DVB-SO_3_H copolymer catalyst. The average glycerol conversion to solketal was about 81% for the fresh catalyst. Subsequent solketal conversions using the regenerated catalyst were 80% for the first recycle, 79% for the second recycle, and 81% for the third recycle. The results demonstrate that the catalyst can be regenerated by treatment with 0.1 M HCl, followed by washing with de-ionized water and drying at 120°C. CNS analysis performed on the fresh and spent ST-DVB-SO_3_H showed no change in composition for the fresh and spent catalysts, with the sulfur content remaining consistent between 8.6 and 8.63 wt.% after three cycles.

These findings are consistent with an existing study, which reported that acid-functionalized ion-exchange resins are reusable (Pico et al., 2013; Eze et al., 2017). Therefore, the synthesized ST-DVB-SO_3_H copolymer catalyst could be an ideal catalyst for continuous solketal production from glycerol by-product in biodiesel processing.

### Techno-Economic Analysis of the Glycerol Acetalization Processes

Techno-economic analysis was used to evaluate the viability of the three process conditions for solketal production as investigated in this study. These solketal process options were one-stage processes at low acetone molar ratio (6:1 of acetone to glycerol molar ratio) to achieve 87% solketal conversion, another one-stage process at higher acetone molar ratio (12:1) to achieve 98% solketal conversion, and a two-stage solketal process using 10:1 of acetone to glycerol molar ratio to obtain 98% solketal conversion. The techno-economic analysis of these three processes using the Aspen (HYSYS) showed that the total capital investment cost for the two-stage process was $27.5 M, $28.7 M for the one-stage process at 12:1 acetone to glycerol molar ratio, and $29.42 M for the one-stage process at 6:1 acetone to glycerol molar ratio (as shown in Table 3).

**Table 3:** Economic evaluation of three different plants to produce solketal from glycerol acetalization (100,000 tons/year).

**Techno-economic parameters**	**One-stage process (12:1 molar ratio)**	**One-stage process (6:1 molar ratio)**	**Two-stage process (10:1 molar ratio)**
TCI ($ /M)	28.7	29.42	27.5
Operating cost ($ /M)	17.2	13.8	15.7
Utility cost ($/M)	14.7	11.4	13.26
Annual net revenue ($/ M)	118.85	67.2	119.36
NPV($/M) at 20 years	703	384	707
Break-even price ($/ton)	2,058	2,088	2,058

As shown in Table 3, the net revenue from the two-stage process was $119.36 M, which was slightly higher than $118.85 M for the one-stage process at 12:1 acetone to glycerol molar ratio, and $67.2 M for the one-stage process at 6:1 acetone to glycerol molar ratio. The NPV for the three glycerol acetalization process options follows similar trends as the net revenue. The NPV of the two-stage process was $707 M, which was higher than $703 M for the one-stage process at 12:1 acetone to glycerol molar ratio, and $384 M for the one-stage process at 6:1 acetone to glycerol molar ratio. This was attributed to the higher cost of the distillation columns that were used in the purification steps of solketal in one-stage process (6:1 of acetone to glycerol molar ratio) to separate solketal from the high amount of glycerol and water. Only one distillation column was used in the purification step of solketal in the one-stage process (12:1 of acetone to glycerol) to separate (98% solketal and 2% of glycerol) from water. No purification step was required in the two-stage process as the formed product was separated from the first step and only a small amount of water (0.16% ≈ 0) was formed in the last stage.

Consequently, the break-even prices of solketal for these processes were reduced to $2,058/ton for the one-stage at 12:1 acetone molar ratio and two-stage process and to $2,088/ton for the one-stage at 6:1 acetone molar ratio, which are all lower than the current price of $3,000/ton for solketal (Alibaba, 2018).

**Figure 11:**
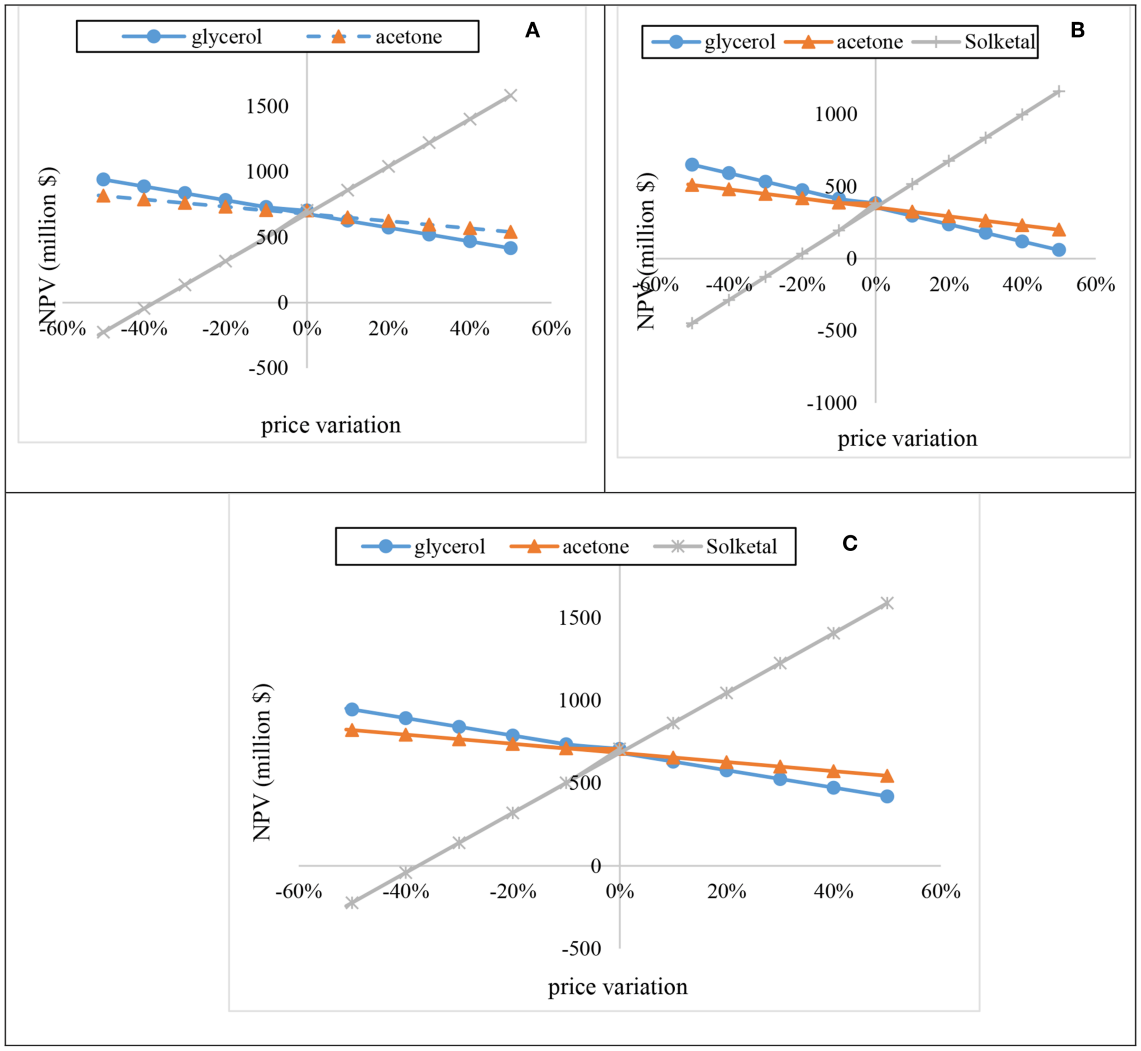
Sensitivity of the process NPV to fluctuations in reactants (acetone and glycerol) and product (solketal) prices.

The sensitivity analysis of acetone, glycerol, and solketal were evaluated by varying their prices from −50 to 50% for 20 years at 15.3% discount rate (Figure 11). As shown in Figures 11A–C, increase in glycerol prices by 10% resulted in decrease in the NPV by about $52.53 M for the one-stage process at 12:1 acetone, $59.18 M for the one-stage process at 6:1 acetone, and $52.53 M for the two-stage process at 10:1 acetone. Increasing the acetone price also had a huge effect on the plant's NPV. For instance, a 10% increase in the acetone price resulted in reductions of the NPV by $27.6 M, $31.1 M, and $ 27.6 M for the one-stage process at 12:1 acetone, one-stage process at 6:1 acetone, and two-stage process at 10:1 acetone, respectively. On the contrary, increasing the solketal price by 10% resulted in a substantial rise in the NPV. As shown in Figures 11A–C, a 10% rise in the solketal price resulted in NPV rise by ~$181 M for one-stage (12:1) and two-stage plants.

## Conclusion

Styrene-divinylbenzene copolymer beads of desired physical properties were synthesized and functionalized for catalytic application in glycerol conversion to solketal. Mixing intensity had a profound effect on the particle size of the copolymer beads, while the DVB and diluent contents determined the degrees of crosslinking and porosity of the beads, respectively. The copolymer beads with the most desirable physical properties (high mechanical stability, porosity, and surface area) were derivatized with sulphonic acid and used in the catalysis of glycerol acetalization with acetone under mild reaction conditions (30–50°C, 2–30 min reaction time, 8.5 wt.% of the catalyst, and 2:1–12:1 acetone to glycerol molar ratio. Both the synthesis of the copolymer beads and the glycerol acetalization processes were investigated using design of experiments (response surface methodology), leading to empirical models for optimization of particle sizes, beads surface area, and swelling ratio. The sulphonic acid-functionalized styrene-divinylbenzene copolymer beads synthesized in this work were found to be catalytically active. This catalyst has similar catalytic activity as the AmberlystTM 70 obtained from Dow Chemical Company, Netherlands. Generally, the glycerol conversion to solketal increased with reaction temperature, residence time, and acetone to glycerol molar ratio.

Three process conditions for glycerol valorization to solketal using a synthesized sulphonic acid-functionalized styrene-divinylbenzene (ST-DVB-SO_3_H) copolymer catalyst were investigated. The ST-DVB-SO_3_H copolymer beads were obtained by copolymerization of 36 wt.% styrene and 64 wt.% divinylbenzene monomer mixture in 84 wt.% diluent based on the total monomer solution, followed by sulphonic acid site functionalization. The ST-DVB-SO_3_H beads produced were spherical of 340 μm mean particles diameter and 2.64 mmol of SO_3_H active sites per gram were produced. Under three processes at 50°C and 8.5 wt.% of ST-DVB: single-stage process (1) 6:1 of acetone to glycerol molar ratio, (2) 12:1 of acetone to glycerol molar ratio to achieve 87% and ≥98% of solketal yield from each process, respectively, and two stages process at 10:1 of acetone to glycerol molar ratio to obtain ≥98%.

Techno-economic analysis was performed using Aspen (HYSYS) for a fixed capacity of 100,000 ton/years and 20-years lifetime, based on the 30-min reaction time. The techno-economic analysis of the three solketal process options at fixed capacity of 100,000 ton/years and 20-years lifetime showed that the net present values for the solketal process options were $707 M for the two-stage, $703 M for the one-stage at 6:1 acetone to glycerol molar ratio, and $384 M for one-stage at 12:1 acetone to glycerol molar ratio.

## Author Contributions

LA-S collected all the data and wrote the first draft of the paper. This work was done under VE and AH supervision.

### Conflict of Interest

The authors declare that the research was conducted in the absence of any commercial or financial relationships that could be construed as a potential conflict of interest.
